# Supplementary material for: The STUbL RNF4 regulates protein group SUMOylation by targeting the SUMO conjugation machinery
Source: Nat Commun. 2017 Nov 27;8:1809. doi: 10.1038/s41467-017-01900-x (PMC5703878; doi:10.1038/s41467-017-01900-x)
Supplement: Supplementary file 1 — Supplementary Information [file 41467_2017_1900_MOESM1_ESM.pdf]

Supplementary Table 1: List of Antibodies.

Antibody	Dilution	Species	Supplier	Cat. No.
Anti-beta-Actin	1/20,000	mouse	Thermo scientific	MA5-15739
Anti-BARD1	1/4000	rabbit	Bethyl	A300-263A
Anti-BARD1	1/1500	rabbit	Santa-Cruz	Sc-11438
Anti-BARD1	1/2000	mouse	Novobiologics	Ab 50984
Anti-BRCA1	1/2000	rabbit	Cell signalling	D54A8
Anti-PIAS1	1/3000	rabbit	Cell Signalling Technology	D33A7
Anti-PIAS1	1/1000	rabbit	Gene Tex	GTX62050
Anti-PIAS4	1/1500	rabbit	Cell Signalling Technology	D2F12
Anti-RNF4	1/2500	rabbit	Eurogentec	Custom made; ref Vyas et al.
Anti-SETDB1 (ESET)	1/1000	mouse	Cell Signalling Technology	2196S
Anti-SUMO2/3	1/20,000	mouse	Abcam	ab81371
Anti-mouse Alexa fluor 488	1/1000	goat	Bethyl	A90-242D2
Anti-mouse Alexa fluor 594	1/1000	goat	Bethyl	A90-242D4

Anti-rabbit Alexa fluor 488	1/500	goat	Thermo-Fisher	35553
Anti-rabbit Alexa fluor 594	1/1000	goat	Thermo-Fisher	35561

Supplementary Table T2: Drugs

**Drugs**	**Supplier**	**Cat no**
Bleocin	Millipore	203401-10MG
MG132	Millipore	474790
DMSO	Sigma	472301
Methyl methanesulfonate	Sigma	129925
Thymidine	Sigma	T9250

Supplementary Table 3: shRNA Constructs

**Target gene**	**TRC No. or ID no.**
RNF4 (I)	TRCN0000017054
RNF4 (II)	TRCN0000272668
RNF4 (III)	TRCN0000284821
PIAS1 (I)	TRCN0000004145
PIAS1 (II)	TRCN0000004147
PIAS4 (I)	TRCN0000004115
PIAS4 (II)	TRCN0000004118
BARD1 (I)	TRCN0000003745
BARD1 (II)	TRCN0000003743
BRCA1 (I)	TRCN0000039837
BRCA1 (II)	TRCN0000039835
None (control shRNA)	SHC002 (Sigma)
None (control shRNA)	Addgene Plasmid # 1864

Supplementary Table T4: List of oligonucleotides.

FW-pCW57.1-stop-rem	CGTTCAGCTTTCTTGTACAAAGTGGTTACCGGTCCACCACCACCAC
RV-pCW57.1-stop-rem	GTGGTGGTGGTGGACCGGTAACCACTTTGTACAAGAAAGCTGAACG
FW-AgeI-C-term-HIS	ACCGGTATGGCTCACCATCACCACCATCATCATCATCATCATTGACCACCACCACCACCACCACT
RV-SpeI-C-terminal-10HIS	ACTAGTGAGACGTGCGGCTTCCG
Fw-AgeI-10HIS-Ubi	ACCGGTATGACTAGCCATCACCATCACCATCACCATCACCATCACTCTAGAGG
Rv-XmaI-Ubi	CCCGGGTCACCCACCTCTGAGACGGAGGA
Rv-XmaI-Ubi-noGlyGly	CCCGGGTCATCTGAGACGGAGGACCAGGT
FW-RNF4-BP	GGGGACAAGTTTGTACAAAAAAGCAGGCTTCATGAGTACAAGAAAGCGTCGTGGT
RV-RNF4-BP	GGGGACCACTTTGTACAAGAAAGCTGGGTCTATATAAATGGGGTGGTACCGTTTGTGG

BARD1_K632R-FW	ATGGATGCTGGATTCTAAGATTTGAATGGGTAAAAGC
BARD1_K632R-RV	GCTTTTACCCATTCAAATCTTAGAATCCAGCATCCAT
BARD1_E634A-FW	TGCTGGATTCTAAAATTTGCATGGGTAAAAGCATGTC
BARD1_E634A-RV	GACATGCTTTTACCCATGCAAATTTTAGAATCCAGCA
BARD1_K96R-FW	GCCTGGATACAAGACTTGAGGATAAATAGACAACTGGAC
BARD1_K96R-RV	GTCCAGTTGTCTATTTATCCTCAAGTCTTGTATCCAGGC
BARD1_K127R-FW	CAGATTTGAAAGAAGATAGACCTAGGAAAAGTTTG
BARD1_K127R-RV	CAAACTTTTCCTAGGTCTATCTTCTTTCAAATCTG
BARD1 L44RFW	TCGCGCCGCGCTCGACCGCAGGGAGAAGCTGCTGCGCTGC
BARD1 L44RW	GCAGCGCAGCAGCTTCTCCCTGCGGTCGAGCGCGGCGCGA
BARD1-Seq1Fw	TGCTACATGACAATGAGCTG
BARD1_Seq1RV	CTTACTTCGAGGGCTAAACCAC
BARD1_Seq2FW	ACGTGGCCATCACAATAGAC
BARD1_seq3FW	TCACCACTTCACGATGCAGCC
BARD1_Seq4FW	AGGCTCAACAGAGAACAGCTG
BARD1 Seq-5	TCTGAGAGAGCCTGTGTGTTTAGG

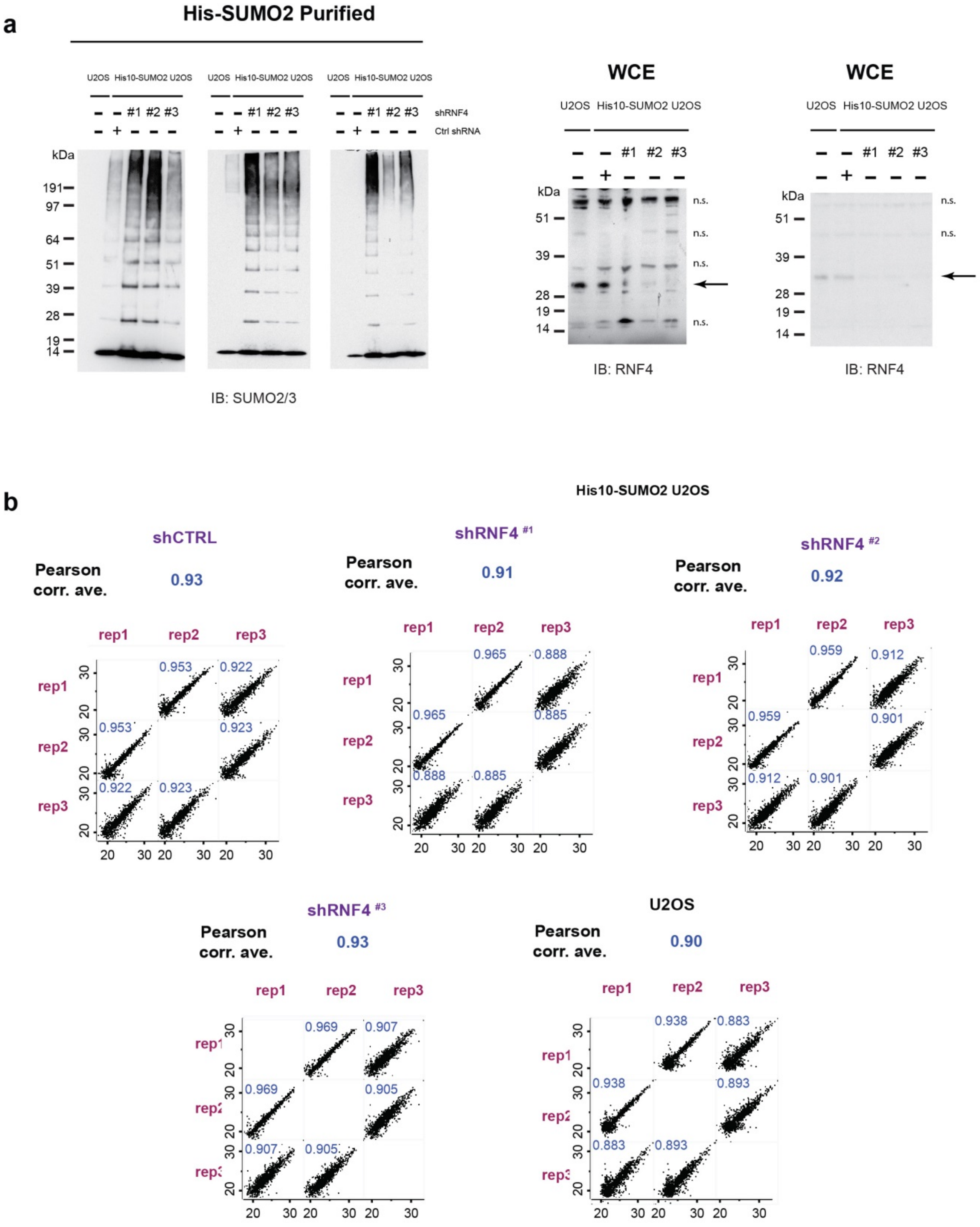

Supplementary Fig. 1

Supplementary figure 1. SUMO2 substrates regulated by the SUMO-Targeted Ubiquitin Ligase (STUbL) RNF4. (**a**) Additional immunoblots are shown for the biological replicates of the experiment presented in Fig. 1c, using antibodies directed against SUMO2/3 and RNF4. n.s. indicates non-specific bands. (**b**) Pearson correlations between the different replicates analysed by mass spectrometry in Fig. 1d.

a

b

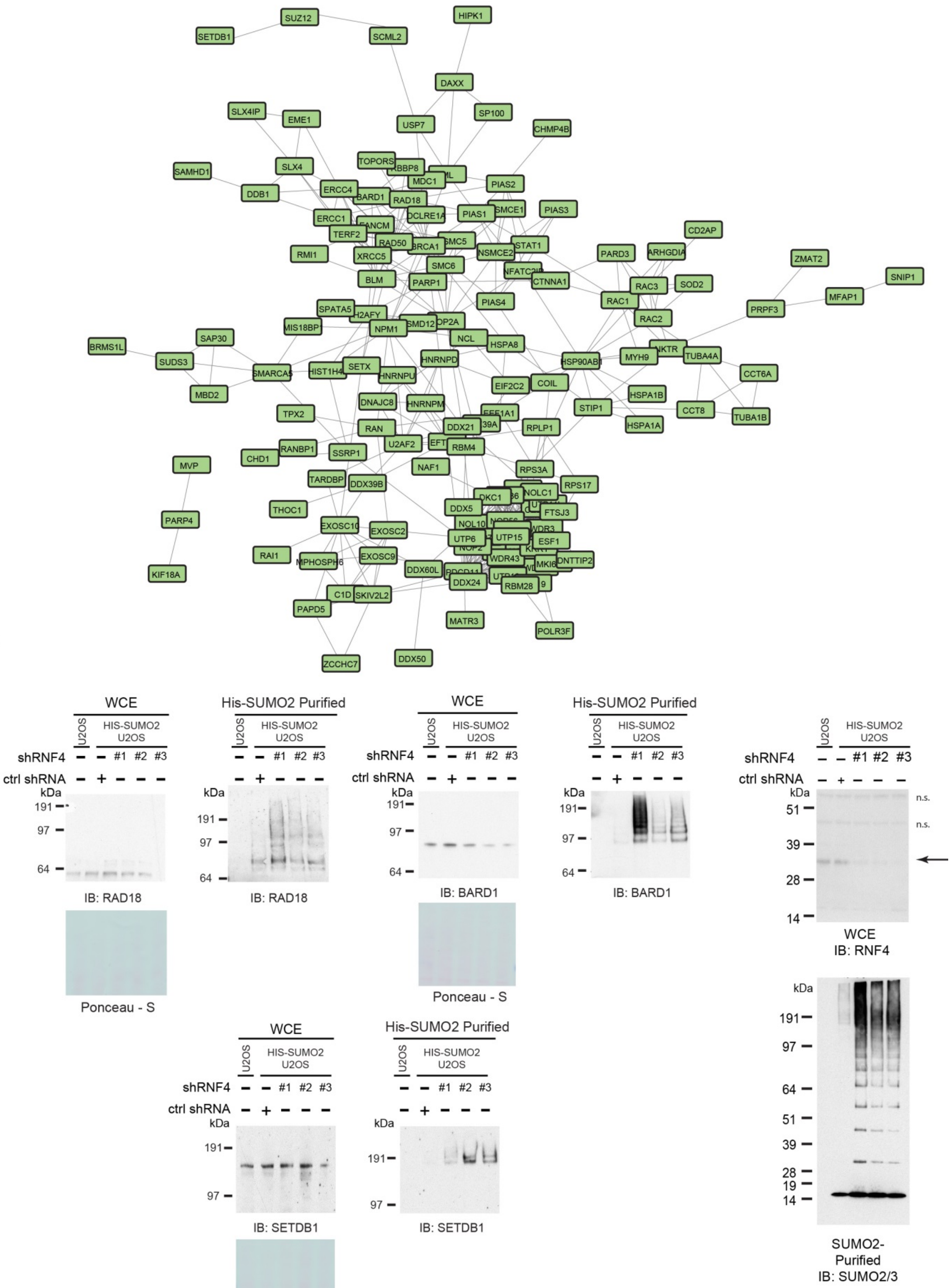

Supplementary Fig. 2

Supplementary figure 2. (**a**) Overview of the RNF4-regulated SUMO2 target protein network. STRING protein interaction network of SUMOylation targets enriched after RNF4 knockdown from Fig. 1d. STRING analysis results were visualized using Cytoscape (version 3.4.0). A high confidence score ($p>0.7$) was used. (**b**) Validation of RNF4 substrates by immunoblotting. U2OS cells stably expressing His10-SUMO2 were separately infected with lentiviruses expressing three different shRNAs directed against RNF4 or a control shRNA. Three days post infection, cells were harvested and His10-SUMO2 conjugates were purified from denaturing lysates. Validated RNF4 substrates include BARD1, SETDB and RAD18. Experiments were independently repeated. RNF4 knockdown efficiency and His10-SUMO2 conjugates were verified by immunoblotting. n.s. indicates non-specific bands.

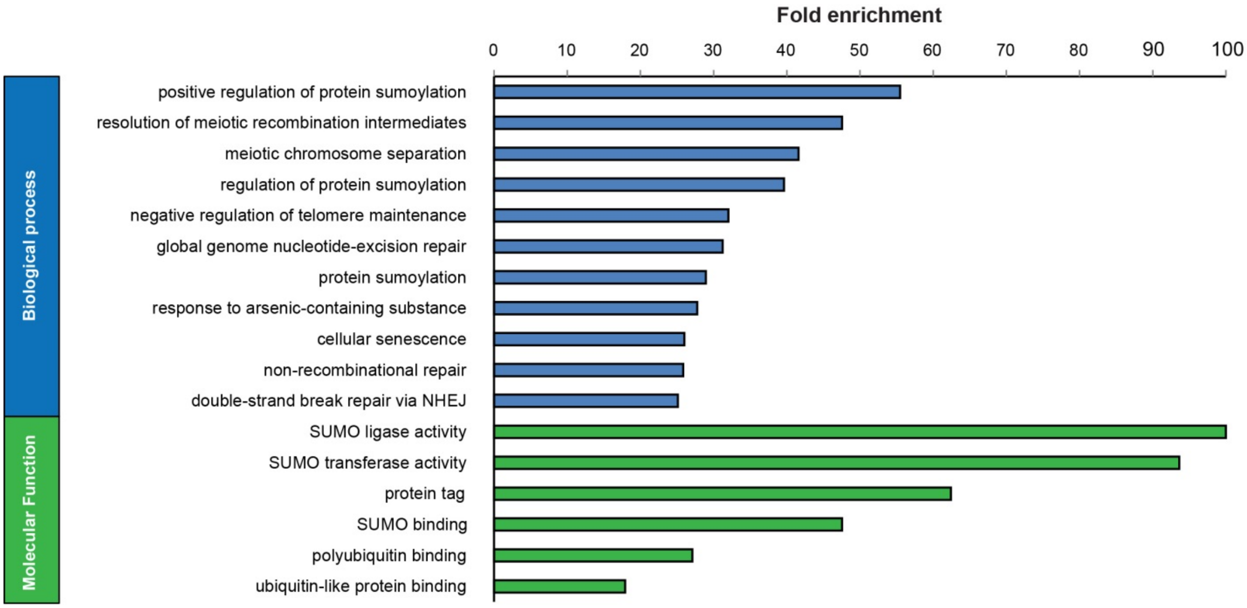

Supplementary Fig. 3

Supplementary figure 3. Gene ontology analysis of all RNF4-TULIP target proteins regarding biological process and molecular function. Full Gene ontology is shown in Supplementary Dataset 5.

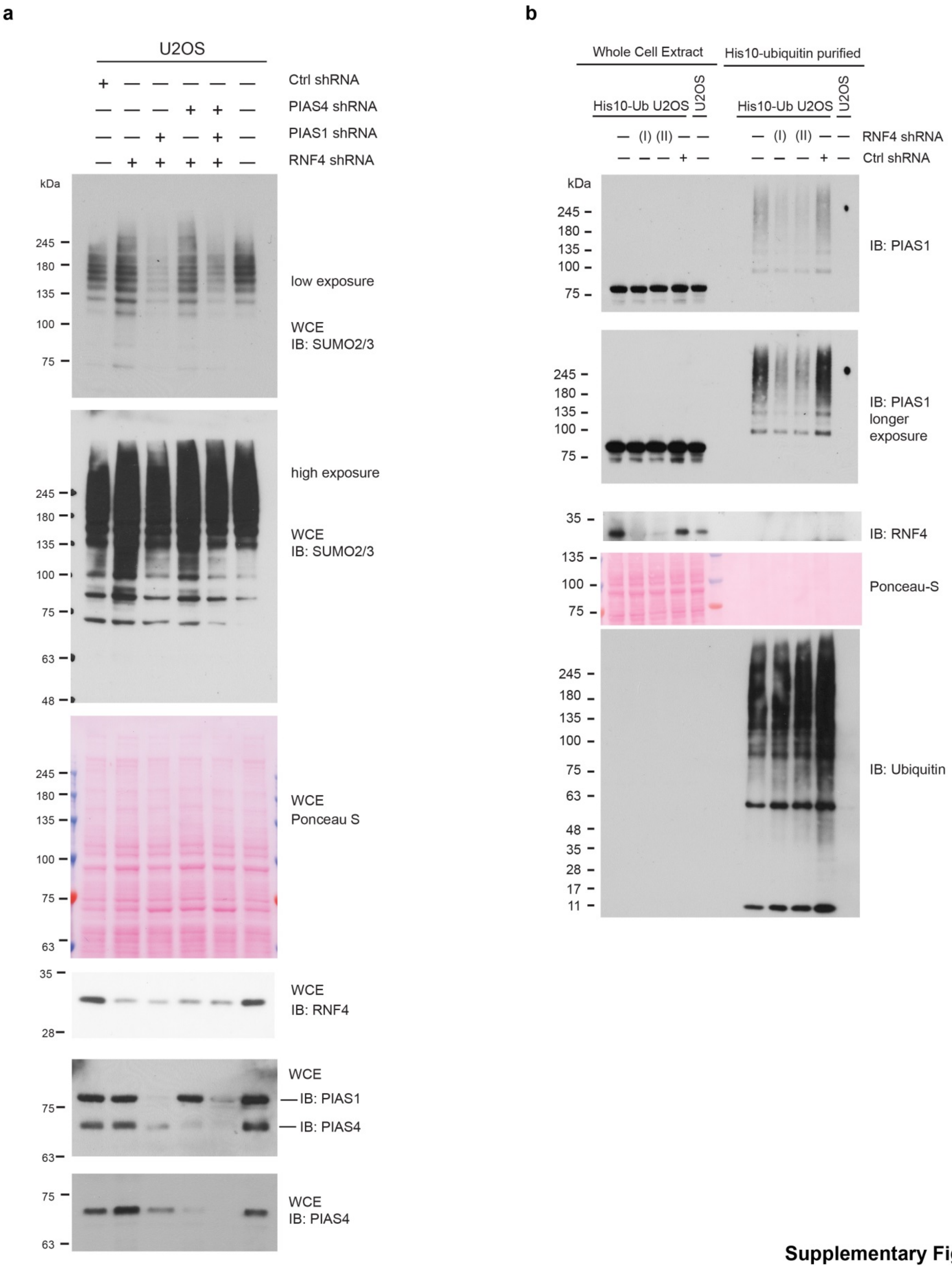

Supplementary Fig. 4

Supplementary figure 4. RNF4 targets the SUMO E3 ligase PIAS1. (**a**) The overall increase in protein SUMOylation upon RNF4 knockdown is counteracted by co-knock down of PIAS1. Independent confirmation of Fig. 4d. U2OS cells were (co)-infected with lentiviruses expressing shRNAs against RNF4, PIAS1 or PIAS4 or a control shRNA as indicated. Three days after infection, cells were lysed in a denaturing buffer and knockdown efficiencies and overall levels of SUMO2/3 were analysed by immunoblotting. (**b**) Independent confirmation of Fig. 4e. RNF4 regulates PIAS1 ubiquitylation. U2OS cells stably expressing His10-ubiquitin were infected with lentiviruses expressing shRNAs directed against RNF4 or a control shRNA. Three days after infection, cells were lysed in a denaturing buffer and His10-ubiquitin conjugates were purified. The levels of ubiquitylated PIAS1 were verified by immunoblotting. Similarly, RNF4 knockdown efficiency was verified by immunoblotting.

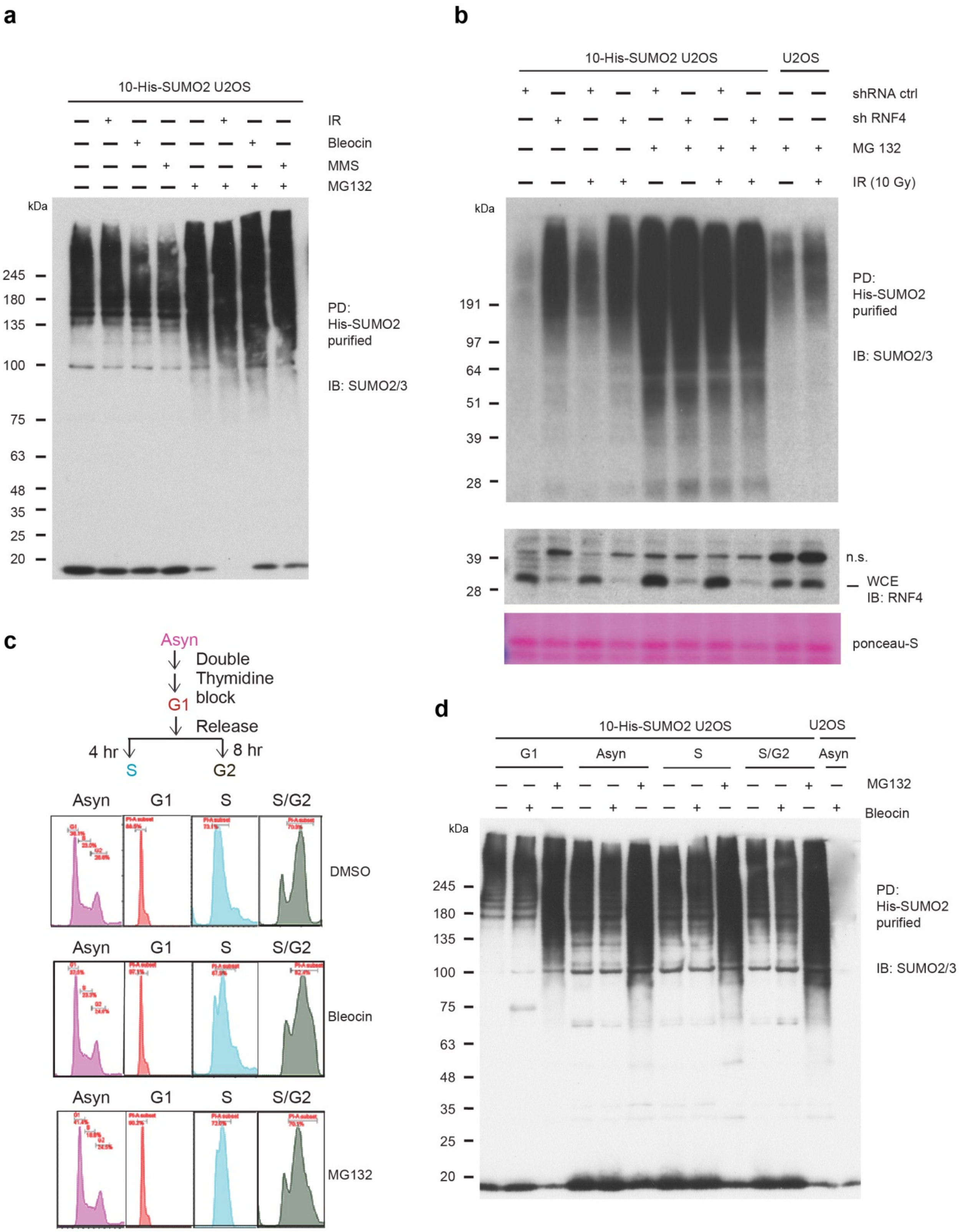

Supplementary Fig. 5

Supplementary figure 5. BARD1 is a RNF4 substrate regulated by DNA damage. (**a**) BARD1 is SUMOylated in response to DNA damage and SUMOylated BARD1 is degraded by the proteasome. Protein samples from the experiment described in Fig. 5a, were immunoblotted with SUMO2/3 antibody. (**b**) Protein samples from the experiment presented in Fig. 5b, were immunoblotted with SUMO2/3 antibody. (**c**) Experimental set up of the cell cycle arrest experiment. To arrest cells at the G1/S boundary, 30% confluent cells were treated with 2 mM thymidine for 19 hrs and then released for 9 hours, followed by a second thymidine (2 mM) block for 17 hrs. G1 arrested cells were washed and released for cell cycle progression by adding fresh cell culture medium. Cells were collected after 4 hours and 8 hours to obtain cells enriched in S-phase or G2/M respectively. FACS profiles of asynchronously growing, G1 arrested, S phase and G2/M cells were determined after propidium iodide (PI) staining. DMSO treated, Bleocin treated and MG132 cells at different stages of cell cycle (b). (**d**) Protein samples from the experiments presented in Fig. 5c and Fig. 5d, were immunoblotted with antibody raised against SUMO2/3. Unprocessed full-size scans of blots are provided in Supplementary Fig. 9.

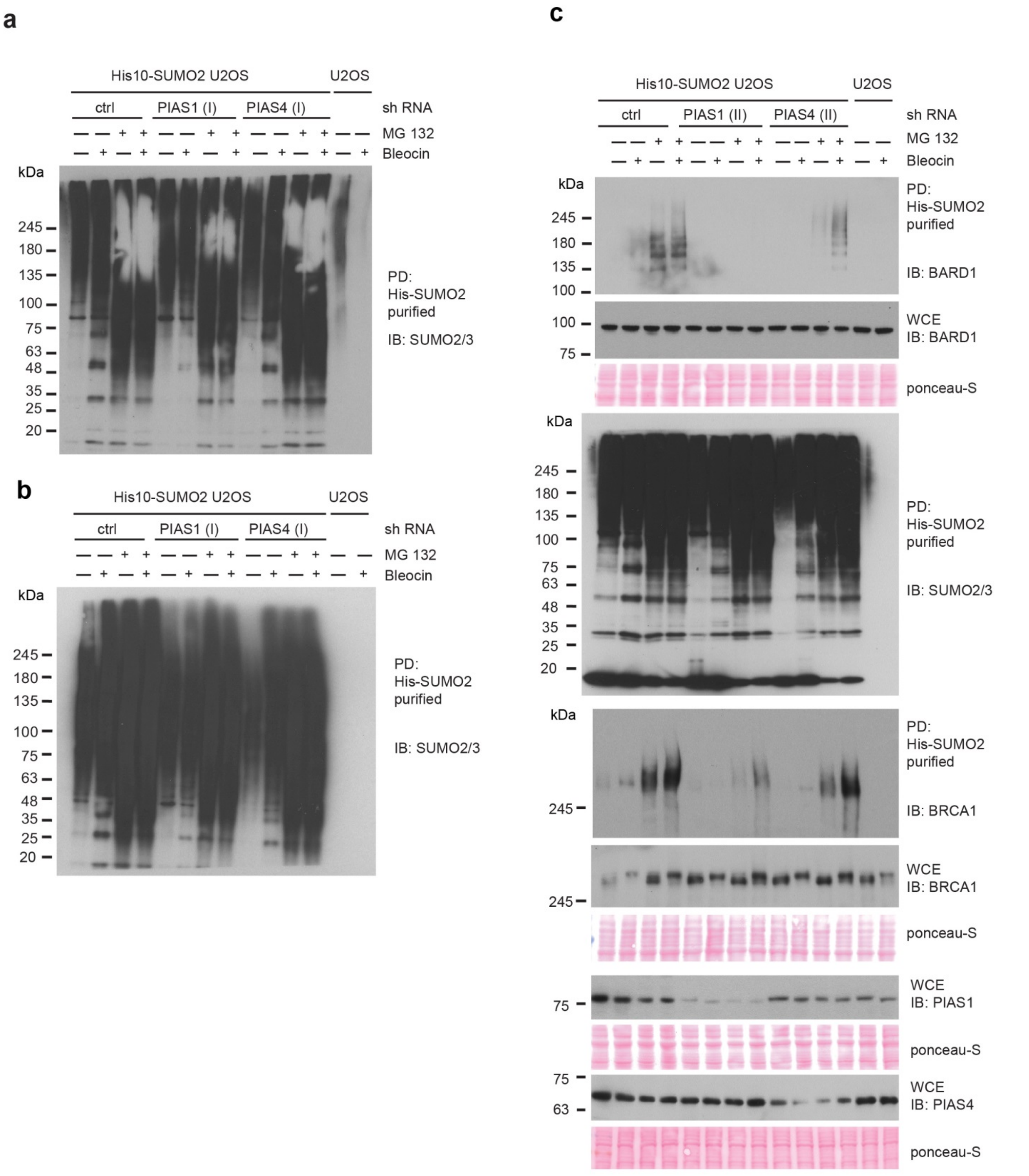

Supplementary Fig. 6

Supplementary figure 6. The SUMO E3 ligase PIAS1, is responsible for the SUMOylation of BARD1 and BRCA1. (**a-b**) Protein samples from experiment presented in Fig. 6a and 6b, were immunoblotted with antibody raised against SUMO2/3. (**c**) The experiment presented in Fig. 6 was repeated with independent sets of shRNAs (PIAS1/II and PIAS4/II) to deplete PIAS1 and PIAS4. Levels of SUMOylated BARD1 and total BARD1 were determined by immunoblotting, Levels of SUMOylated BRCA1 and total BRCA1 were determined by immunoblotting. Knockdown efficiencies of PIAS1 and PIAS4 were determined by immunoblotting. Additionally, the SUMO2 purification efficiency was determined by immunoblotting. Unprocessed full-size scans of blots are provided in Supplementary Fig. 9.

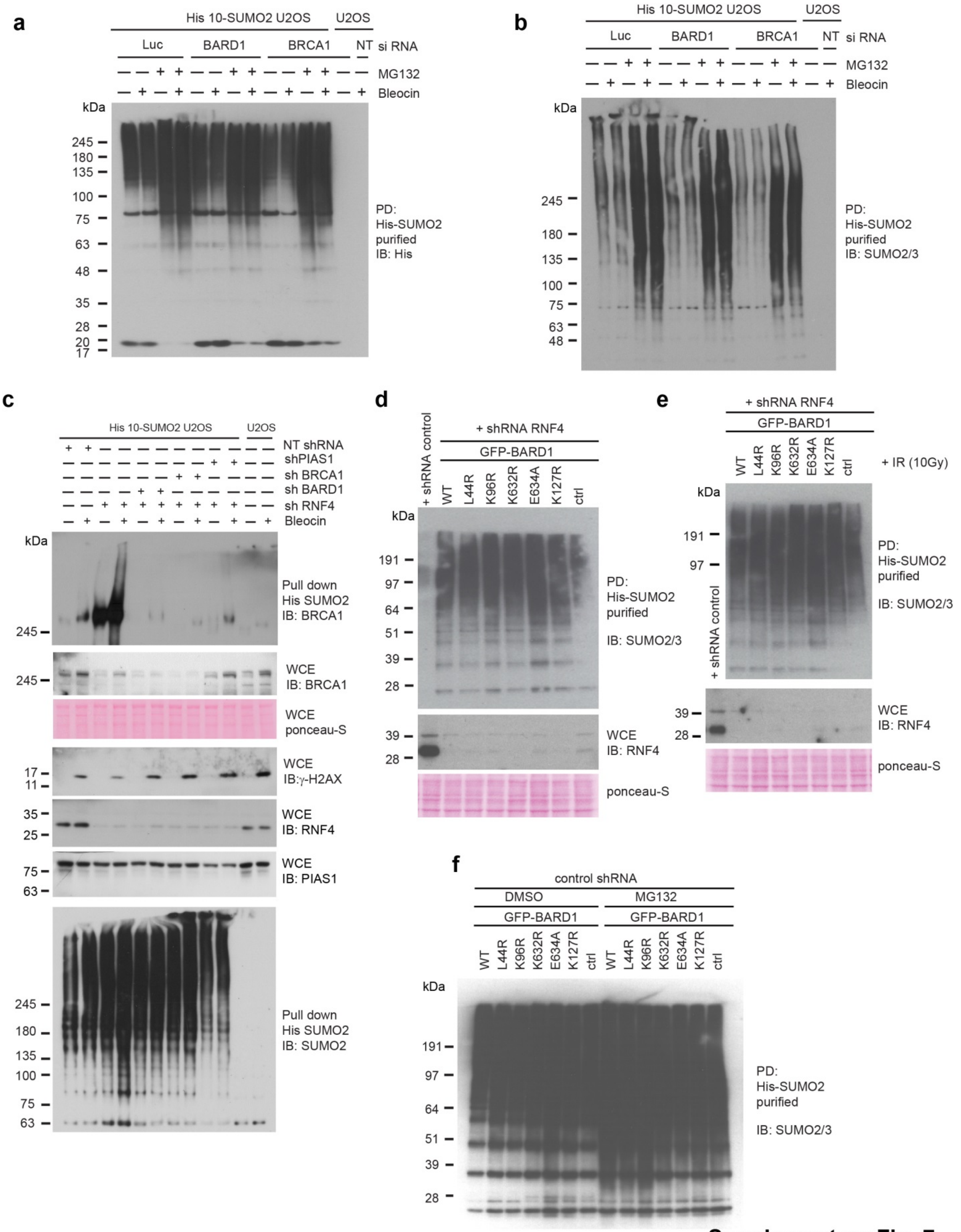

Supplementary Fig. 7

Supplementary figure 7. SUMOylation of BARD1 occurs in a BRCA1-dependent manner. (**a-b**). Protein samples from experiments presented in Fig. 7a and 7b were immunoblotted with antibody raised against SUMO2/3. (**c**) Protein samples from the experiment presented in Fig. 7b were immunoblotted with antibody raised against SUMO2/3. To determine the RNF4 depletion level whole cell extracts were immunoblotted with RNF4 antibody. To determine de formation of DNA damage whole cell extracts were immunoblotted against γ-H2A.X (**d-f**) Protein samples from experiment presented in Fig. 7(c-e) respectively, were immunoblotted with SUMO2/3 antibody. Unprocessed full-size scans of blots are provided in Supplementary Fig. 9.

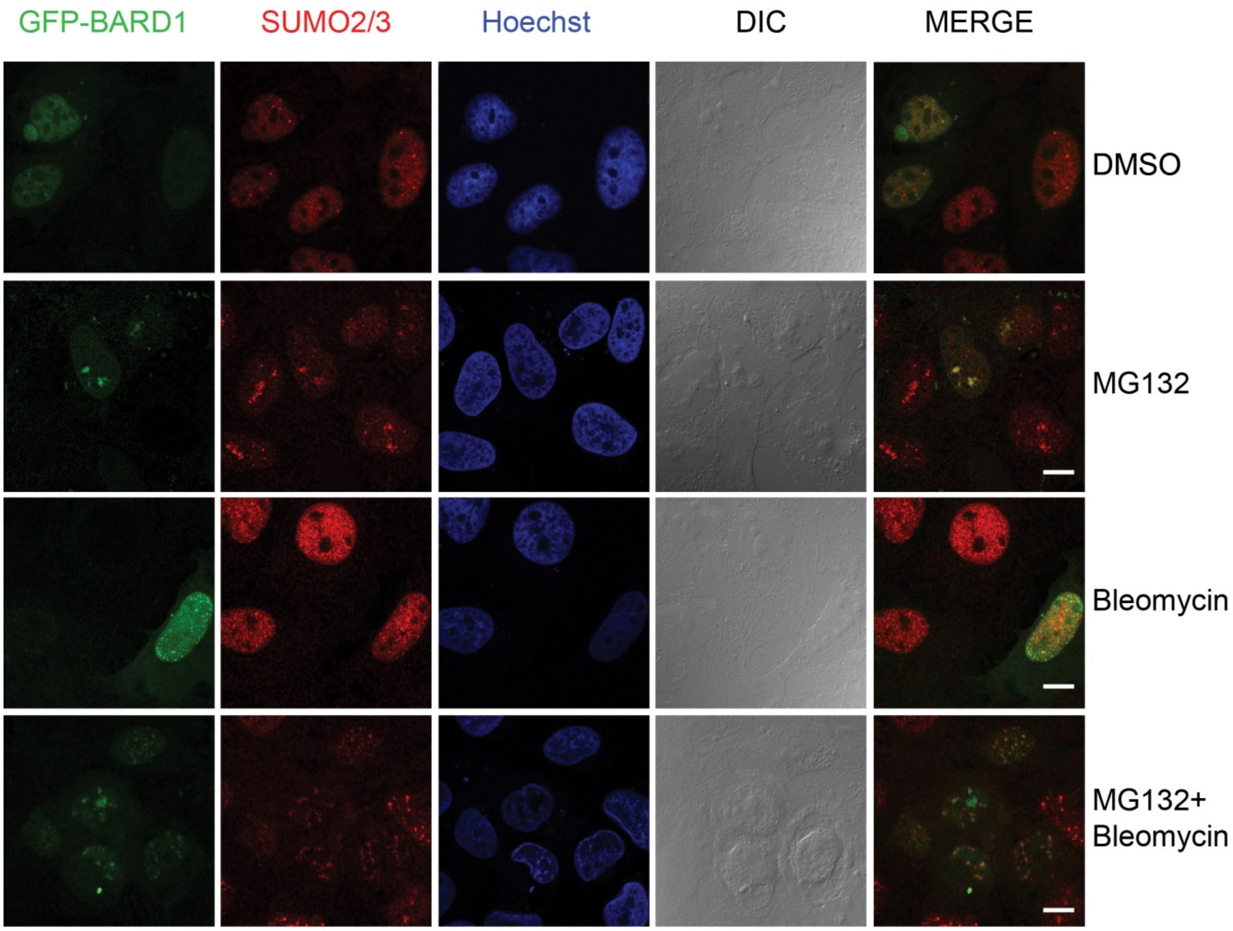

Supplementary Fig. 8

Supplementary figure 8. Co-localization of BARD1 and SUMO2/3 in response to DNA damage and proteasome inhibition. Independent biological replicate of the experiment presented in Fig. 8.

all full scale scans for immunoblots related to all figures

WCE

---

related to Fig. 1c

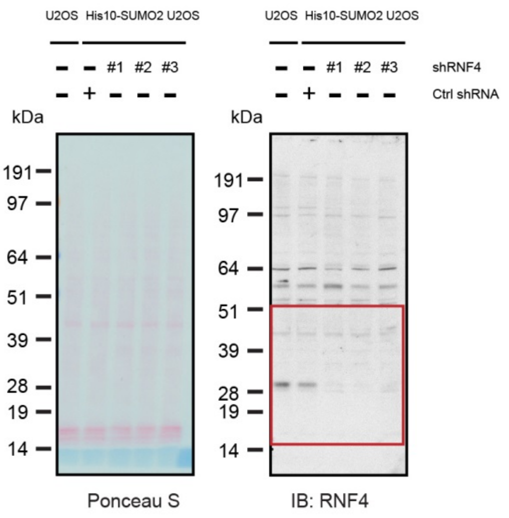

related to Supplementary Fig. 1a

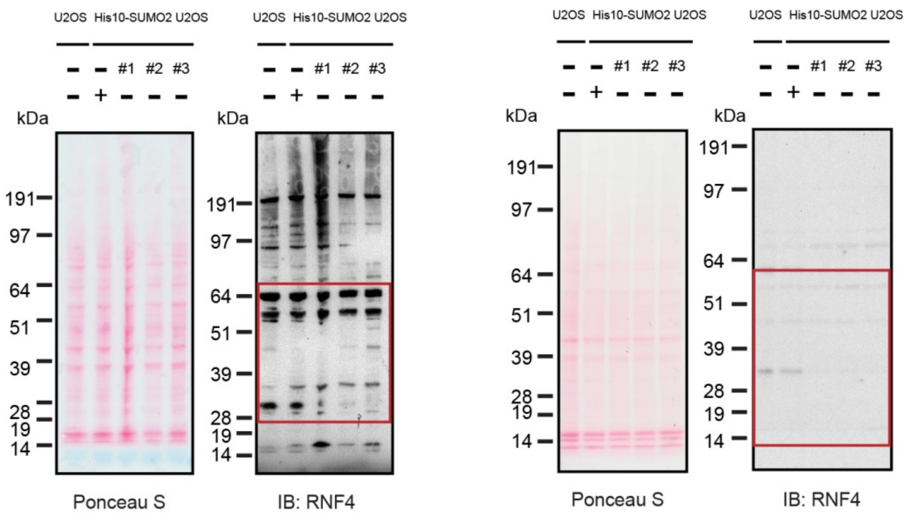

Supplementary Fig. 9

all full scale scans for immunoblots related to all figures

related to Supplementary Fig. 2b

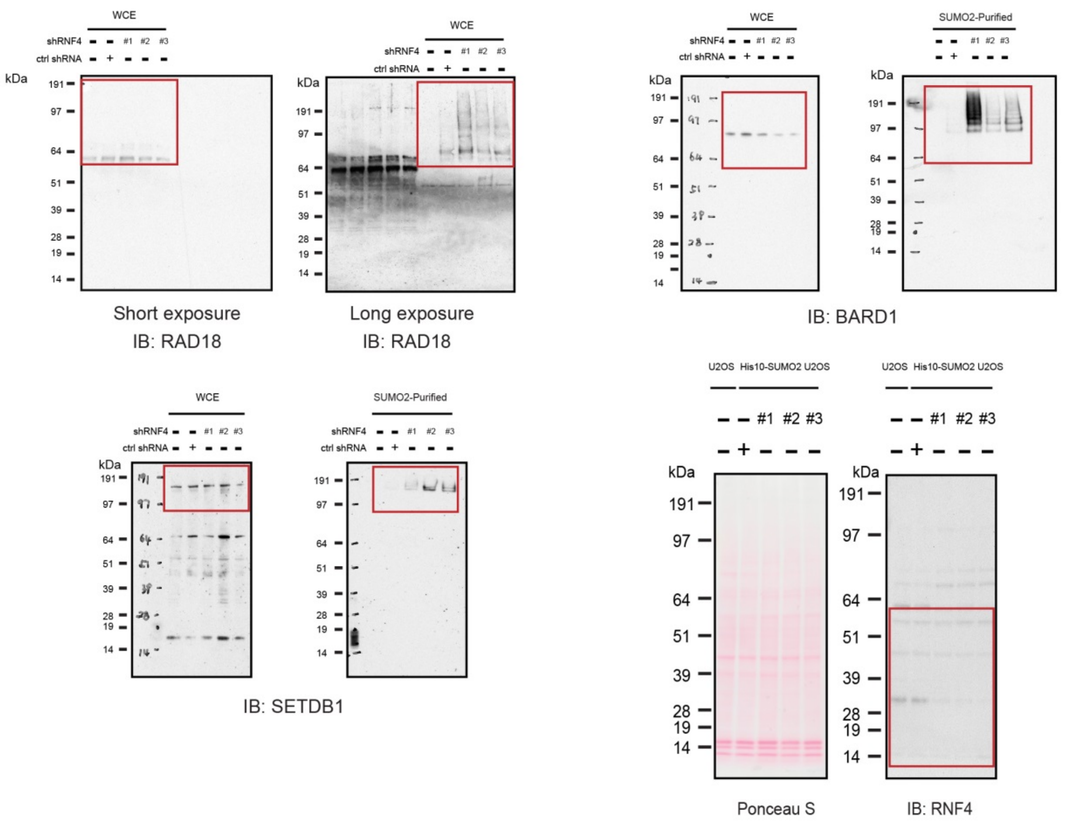

Supplementary Fig. 9

all full scale scans for immunoblots related to all figures

related to Fig. 3a

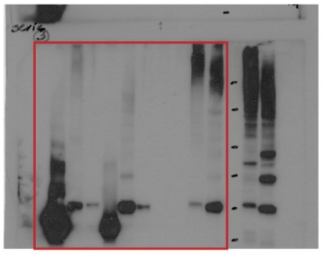

related to Fig. 4b

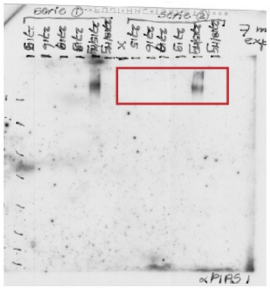

related to Fig. 4c

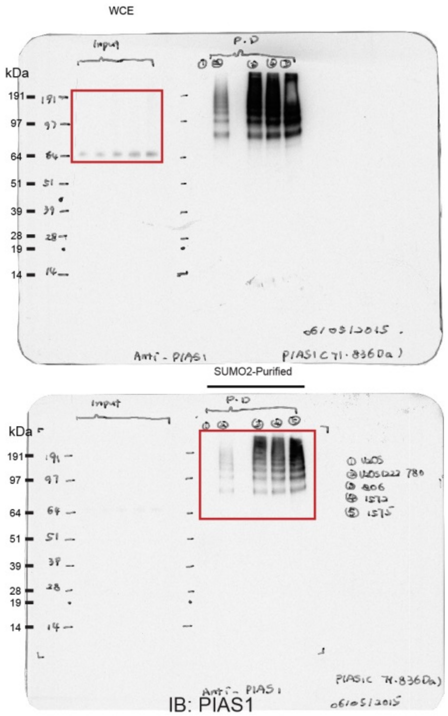

related to Fig. 4d

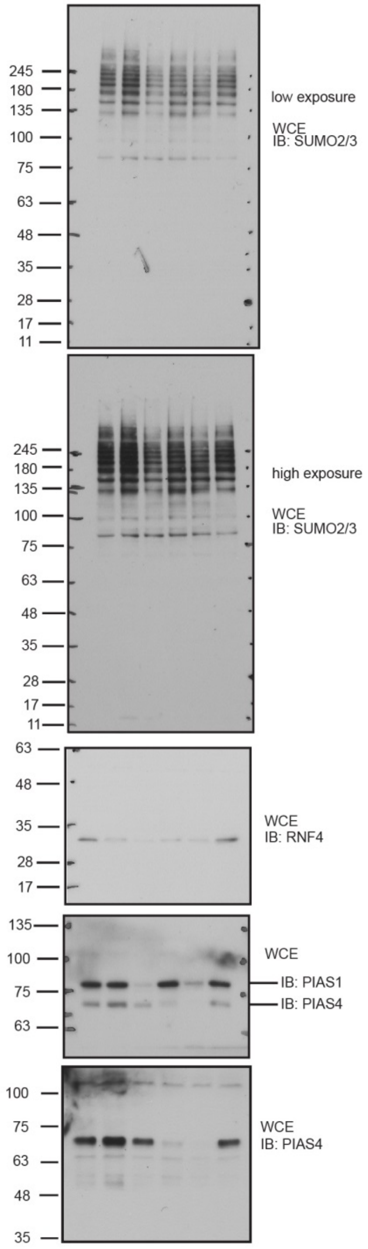

related to Fig. 4e

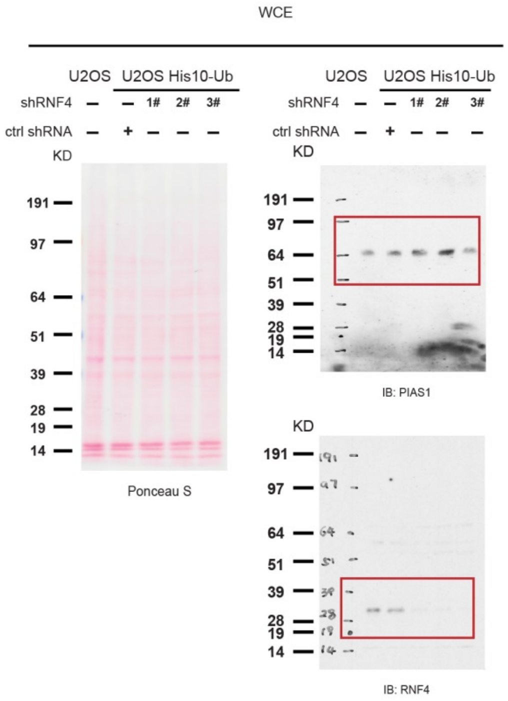

Supplementary Fig. 9

all full scale scans for immunoblots related to all figures

related to Fig. 5a / Supplementary Fig. 5a

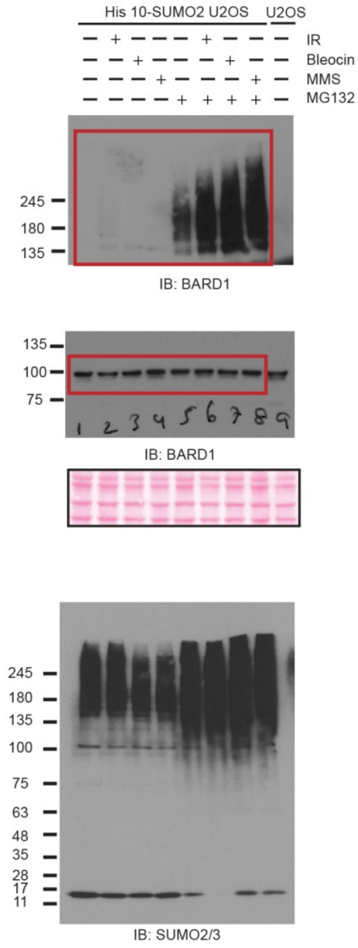

related to Fig. 5b / Supplementary Fig. 5b

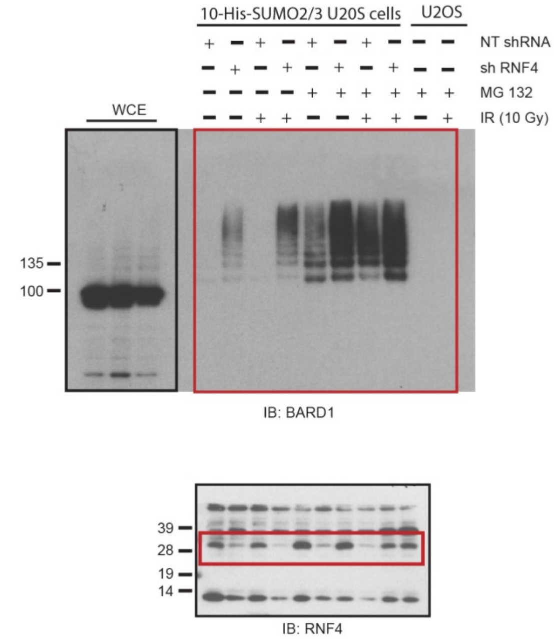

related to Fig. 5d

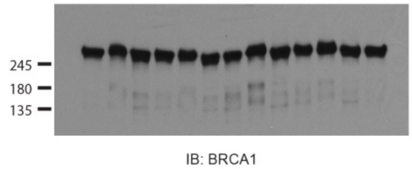

related to Fig. 5c

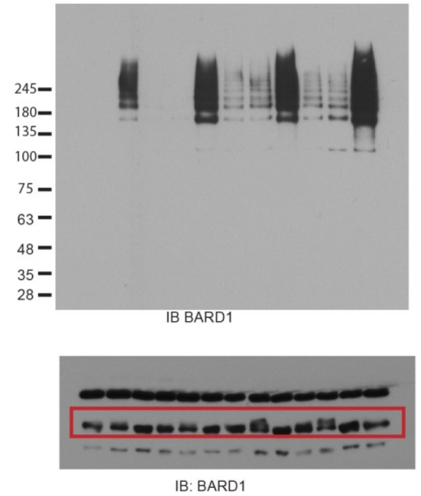

Supplementary Fig. 9

all full scale scans for immunoblots related to all figures

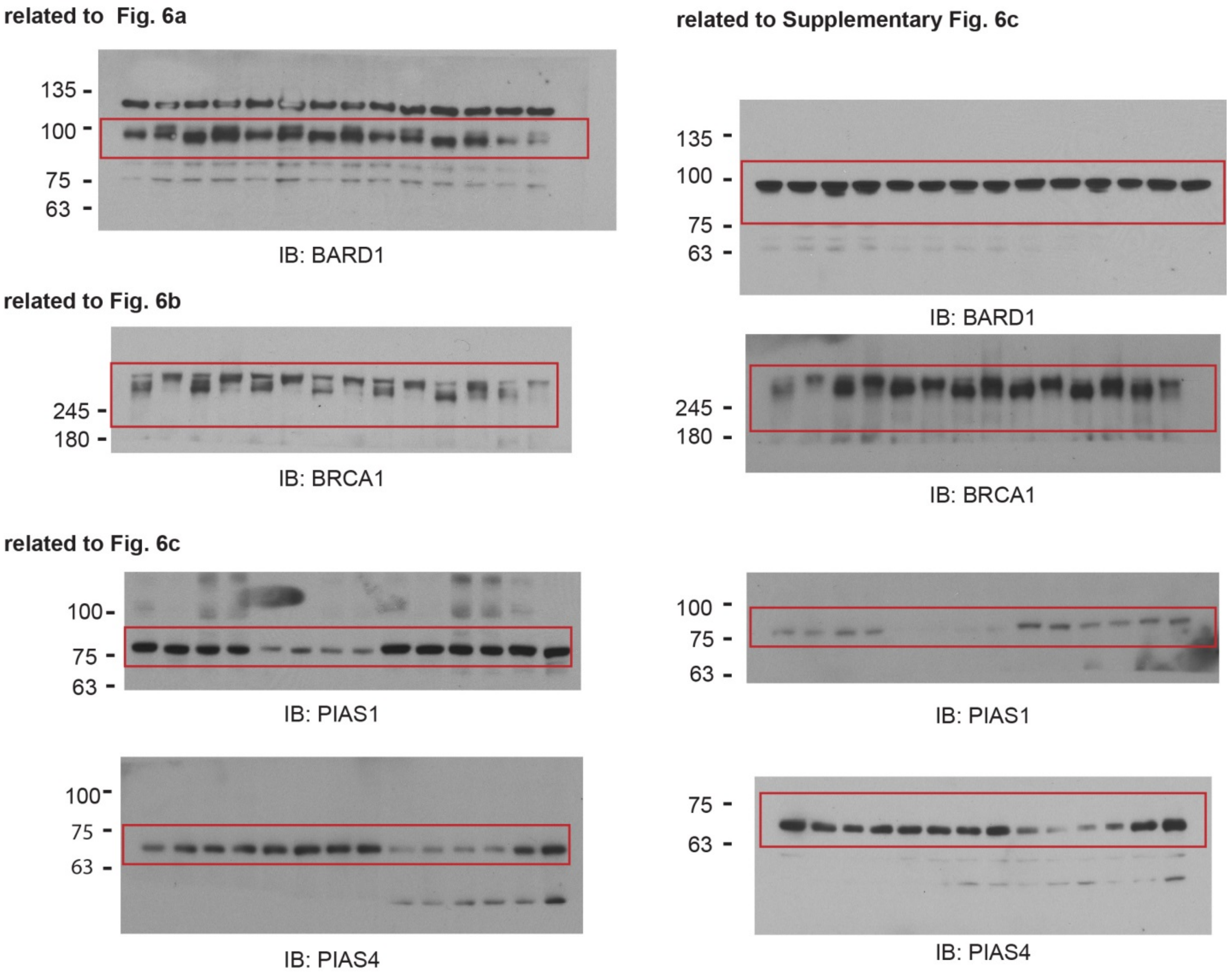

Supplementary Fig. 9

all full scale scans for immunoblots related to all figures

related to Fig. 7a

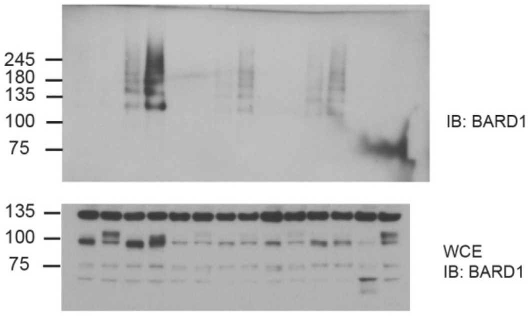

related to Fig. 7b.

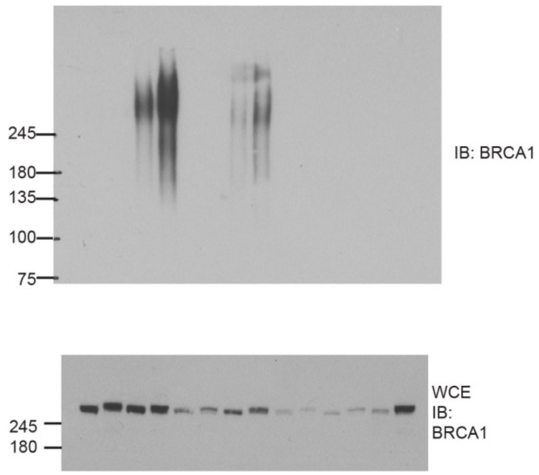

related to Supplementary Fig. 7c

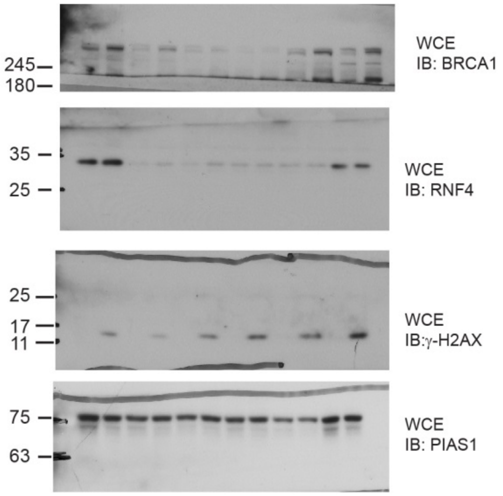

related to Supplementary Fig. 7d

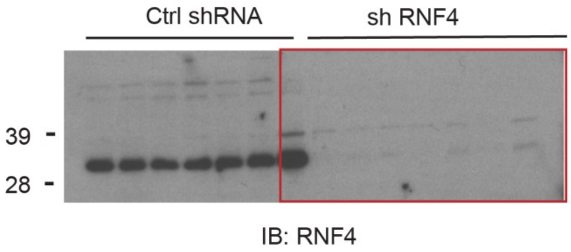

related to Supplementary Fig. 7e

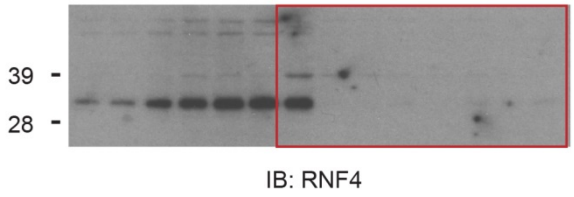

Supplementary Fig. 9
