# Supplementary material for: The STUbL RNF4 regulates protein group SUMOylation by targeting the SUMO conjugation machinery
Source: Nat Commun. 2017 Nov 27;8:1809. doi: 10.1038/s41467-017-01900-x (PMC5703878; doi:10.1038/s41467-017-01900-x)
Supplement: Supplementary file 2 — Descriptions of Additional Supplementary Files [file 41467_2017_1900_MOESM2_ESM.pdf]

Descriptions of Additional Supplementary Information

File Name: Supplementary Dataset 1
Description: SUMO enriched after RNF4 knockdown. A complete list of proteins identified in our RNF4 knockdown project, including gene names, whether they are SUMO targets or not and if they are enriched after RNF4 knockdown. Average enrichment, p-values, and other relevant information is provided.

File Name: Supplementary Dataset 2
Description: . Gene ontology of SUMO targets enriched after RNF4 knockdown. Complete gene ontology study of the SUMOylation targets enriched after RNF4 knockdown. The data were generated using the PANTHER overrepresentation test from the Gene Ontology Consortium.

File Name: Supplementary Dataset 3
Description: RNF4-TULIP targets. Complete list of proteins identified in our RNF4-TULIP project. Gene and protein names are provided and whether they are RNF4-TULIP targets or not in different conditions. Average enrichment, p-values and other relevant information is provided.

File Name: Supplementary Dataset 4
Description: RNF4-knockdown and -TULIP targets. Combined table showing proteins enriched after RNF4 knockdown and/or in the TULIP assay. Included is enrichment information in different conditions and whether they are SUMOylation targets according to Hendriks et al. 2014.

File Name: Supplementary Dataset 5
Description: Gene ontology of RNF4-TULIP targets. Complete gene ontology study of the RNF4-TULIP targets. Data were generated using the PANTHER overrepresentation test from the Gene Ontology Consortium.
